# Increased transcriptional activity of prostate-specific antigen in the presence of TNP-470, an angiogenesis inhibitor

**DOI:** 10.1038/sj.bjc.6690253

**Published:** 1999-03

**Authors:** J Horti, S C Dixon, C J Logothetis, Y Guo, E Reed, W D Figg

**Affiliations:** Medicine Branch, Division of Clinical Sciences, National Cancer Institute, Bldg. 10, Bethesda, Maryland, 20892, USA; GU Oncology, Department of Medical Oncology,The University of Texas MD Anderson Cancer Center, Houston, Texas, 77030, USA

**Keywords:** prostate-specific antigen, androgen-independent prostate cancer, TNP-470, AGM-1883, androgen receptor

## Abstract

Prostate-specific antigen, PSA, is regarded as a reliable surrogate marker for androgen-independent prostate cancer (AIPC). Concern has been raised that investigational agents may affect PSA secretion without altering tumour growth or volume. In a phase I trial, several patients with AIPC had elevated serum PSA levels while receiving TNP-470 that reversed upon discontinuation. TNP-470 inhibits capillary growth in several angiogenesis models. These observations prompted us to determine if TNP-470, or its metabolite, AGM-1883, altered PSA secretion. Intracellular protein and transcriptional levels of PSA and androgen receptor were also determined. The highest TNP-470 concentration produced a 40.6% decrease in cell number; AGM-1883 had minimal effects on cell viability. PSA secretion per cell was induced 1.1- to 1.5-fold following TNP-470 exposure. The same trend was observed for AGM-1883. PSA and AR were transcriptionally up-regulated within 30 min after exposure to TNP-470. PSA transcription was increased 1.4-fold, while androgen receptor (AR) transcription was induced 1.2-fold. The increased PSA transcriptional activity accounts for the increased PSA secretion. Increased AR transcription was also reflected at the protein level. In conclusion, TNP-470 and AGM-1883 both up-regulated PSA making clinical utilization of this surrogate marker problematic. © 1999 Cancer Research Campaign


*British Journal of Cancer* (1999) **79**(9/10), 1588–1593
© 1999 Cancer Research Campaign
Article no. bjoc.1998.0253

Short communication

### Increased transcriptional activity of prostate-specific antigen in the presence of TNP-470, an angiogenesis inhibitor

**J Horti[1,*], SC Dixon[1,*], CJ Logothetis[2], Y Guo[1], E Reed[1] and WD Figg[1]**

[1]Medicine Branch, Division of Clinical Sciences, National Cancer Institute, Bldg. 10, Rm 5A01, Bethesda, Maryland 20892, USA; [2]GU Oncology, Department of Medical Oncology, The University of Texas MD Anderson Cancer Center, Houston, Texas 77030, USA

**Summary** Prostate-specific antigen, PSA, is regarded as a reliable surrogate marker for androgen-independent prostate cancer (AIPC). Concern has been raised that investigational agents may affect PSA secretion without altering tumour growth or volume. In a phase I trial, several patients with AIPC had elevated serum PSA levels while receiving TNP-470 that reversed upon discontinuation. TNP-470 inhibits capillary growth in several angiogenesis models. These observations prompted us to determine if TNP-470, or its metabolite, AGM-1883, altered PSA secretion. Intracellular protein and transcriptional levels of PSA and androgen receptor were also determined. The highest TNP-470 concentration produced a 40.6% decrease in cell number; AGM-1883 had minimal effects on cell viability. PSA secretion per cell was induced 1.1- to 1.5-fold following TNP-470 exposure. The same trend was observed for AGM-1883. PSA and AR were transcriptionally up-regulated within 30 min after exposure to TNP-470. PSA transcription was increased 1.4-fold, while androgen receptor (AR) transcription was induced 1.2-fold. The increased PSA transcriptional activity accounts for the increased PSA secretion. Increased AR transcription was also reflected at the protein level. In conclusion, TNP-470 and AGM-1883 both up-regulated PSA making clinical utilization of this surrogate marker problematic.

**Keywords:** prostate-specific antigen; androgen-independent prostate cancer; TNP-470; AGM-1883; androgen receptor

Prostate-specific antigen (PSA), a 33 kDa glycoprotein, is regarded as a reliable surrogate marker for survival and disease progression in patients with androgen-independent prostate cancer (AIPC) (Sridhara et al, 1995). Approximately 90% of patients with advanced metastatic prostate cancer have an elevated PSA (Figg et al, 1996). Furthermore, by multivariant analysis, PSA level is a predictor of clinical outcome. Kelly and colleagues found a median survival of > 25 months in those patients exhibiting a greater than 50% decline in PSA following an investigational regimen versus 8.6 months in those patients not achieving that level (Kelly et al, 1993). Our group reported a median survival of 19.0 versus 6.3 months in patients with AIPC that experienced a 50% PSA decline versus those that did not (Thibault et al, 1993).

Concern has been raised that some investigational agents may affect PSA production or secretion without altering tumour growth or volume. Phenylacetate, α-interferon and *cis*-retinoic acid have been shown to up-regulate PSA expression (Walls et al, 1996). These are differentiation agents and may effect PSA through that mechanism. However, carboxy-amidotriazole, an antimetastatic, antiproliferative agent, has been reported to down-regulate PSA expression (Wasilenko et al, 1996). Suramin also has been reported to inhibit PSA expression (Thalmann et al, 1996). Thus, the utilization of PSA as a surrogate marker is questionable without first evaluating the effect of new investigational drugs on PSA production.

Proliferation of new blood vessels is essential to continued growth of solid tumours and inhibition of angiogenesis has been proposed as a potential means for selectively impairing tumour growth (Folkman and Ingber, 1992). Compounds that inhibit angiogenesis include protamine (Taylor and Folkman, 1982), interferon (Voest et al, 1995), specific steroids in combination with heparin (Thorpe et al, 1993), a sulfated polysaccharide–peptidoglycan complex (Inuoe et al, 1988), pentosan (Lush et al, 1996), thalidomide (Figg et al, 1997*a*), platelet factor-4 (Maione et al, 1990) and D-penicillamine (Matsubara et al, 1989). A new class of compounds was discovered when a fungal contaminant of a capillary endothelial cell culture was observed to produce a zone of endothelial cell rounding, a phenomenon characteristic of other antiangiogenic compounds (Ingber et al, 1990). The substance responsible for this phenomenon was fumagillin, an antibiotic naturally secreted by the fungus *Aspergillus fumigatus*. Although fumagillin had anti-angiogenic activity both in endothelial cell cultures and in vivo, it caused severe weight loss in mice with prolonged administration. A search to identify less toxic analogues yielded TNP-470 which was 50 times more potent than fumagillin in inhibiting capillary growth and was less toxic.

One possible mechanism for the effect of TNP-470 on angiogenesis is inhibition of growth factor-induced DNA synthesis in endothelial cells. TNP-470 potently inhibits endothelial cell growth and cytotoxicity occurs at concentrations greater than 1 μg $ml^{-1}$ (Kusaka et al, 1991; Kato et al, 1994). The angioinhibitory activity appears to involve transcriptional inhibition of specific cdk and

*Received 10 February 1998*
*Revised 16 June 1998*
*Accepted 10 August 1998*

*Correspondence to*: WD Figg, Medicine Branch, Division of Clinical Sciences, National Cancer Institute, 10 Center Drive, Bldg 10, Room 5A01, Bethesda, MD 20892, USA

*Both Dr Horti and Dr Dixon should be viewed as sharing first authorship.

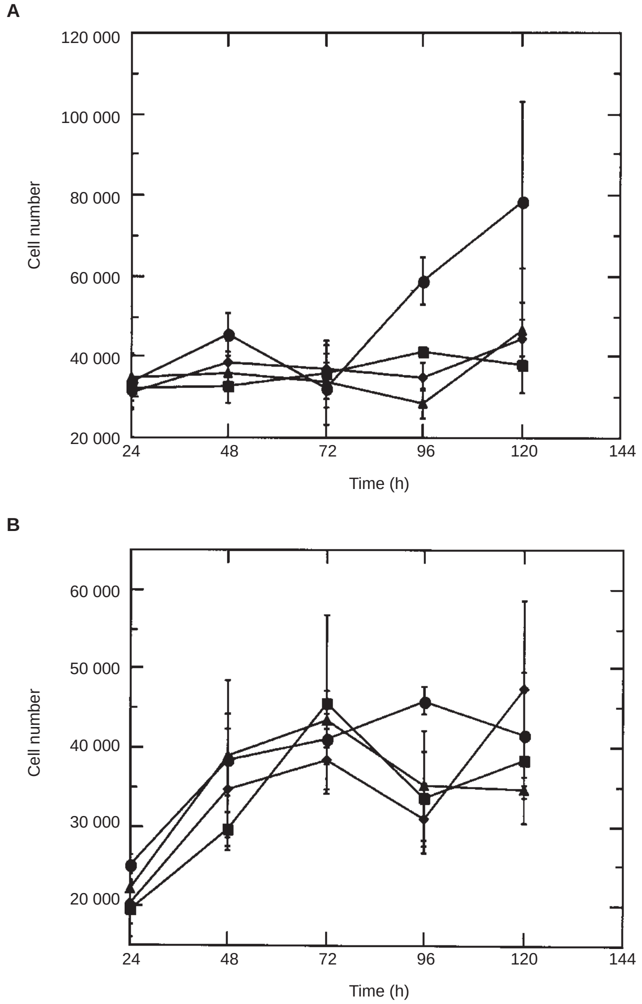

**Figure 1** The number of LNCaP cells determined every 24 h following initial exposure to three concentrations of either (**A**) TNP-470 or (**B**) AGM-1883. Each result is the mean of the cell counts obtained from three wells (±SD). The (●) represent control wells; (■) were treated at 50 ng ml$^{-1}$; (◆) were treated at 500 ng ml$^{-1}$; and (▲) were treated at 1000 ng ml$^{-1}$

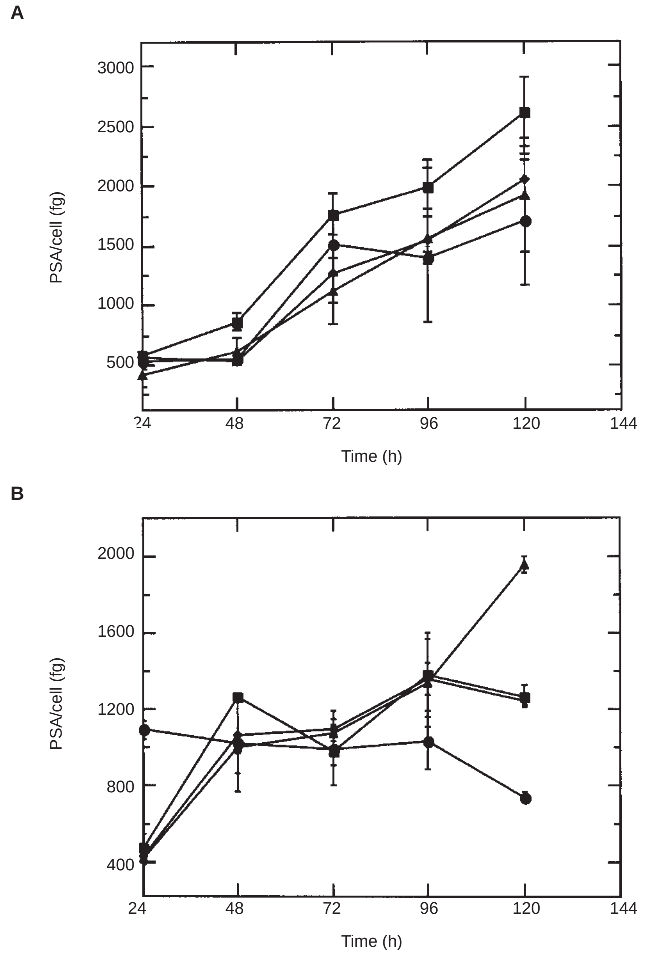

**Figure 2** The amount of PSA (fg) normalized to the cell number every 24 h following an initial exposure to either (**A**) TNP-470 or (**B**) AGM-1883. Each value represents the mean of the PSA values measured for each well divided by the corresponding cell number (± SD) normalized to control cyclin gene family members. TNP-470 may also inhibit cdc2 and cdk2 kinase activation in endothelial cells (Kato et al, 1994). Alteration of thrombospondin production, an important regulator of endothelial cell proliferation and angiogenesis, is another proposed mechanism of action (Abe et al, 1994; Kato et al, 1994).

Kusaka and associates demonstrated inhibition of in vitro capillary growth and induction of avascular zones in the chick chorioallantoic membrane assay (Kusaka et al, 1991). A rat corneal micropocket assay showed 20 μg TNP-470 per cornea suppressed the number and length of blood vessels induced by basic fibroblast growth factor (bFGF). In the sponge implantation model in rats, systemic administration of TNP-470 inhibited angiogenesis induced by bFGF. Finally, in the rat blood vessel organ culture assay, 1 ng ml$^{-1}$ TNP-470 inhibited capillary-like tube formation (Kusaka et al, 1991; Bauer et al, 1998). TNP-470 also inhibited solid tumour growth in animal models (Ingber et al, 1990; Kusaka et al, 1991; Kato et al, 1994; O'Reilly et al, 1995). Ingber and colleagues reported that 30 mg kg$^{-1}$ TNP-470 every other day inhibited the growth of Lewis lung carcinoma, B16 melanoma, and other mouse tumours (Ingber et al, 1990). In some studies, tumour inhibition was noted with administration as infrequently as once a week. The dose-limiting toxicity of TNP-470 in dogs was cerebral bleeding when high doses were given as a bolus (Masiero et al, 1997).

Logothetis et al have completed a phase I study of TNP-470 in patients with AIPC (Logothetis et al, 1997). They reported a maximum tolerated dose of 70.88 mg m$^{-2}$ when a 1 h intravenous infusion was administered every other day for 28 days, repeated every 6 weeks. In this trial, several patients had serum PSA elevations while receiving active therapy that reversed upon discontinuation. Sartor reported a TNP-470 withdrawal phenomenon in a single patient with metastatic prostate cancer (Sartor, 1995). Based on these observations, we wanted to determine if TNP-470, or its metabolite, AGM-1883, alters PSA secretion in an in vitro system. Furthermore, we wanted to determine if protein expression or transcription was altered in LNCaP cells exposed to clinically achievable concentrations of either of these compounds.

*© Cancer Research Campaign 1999*

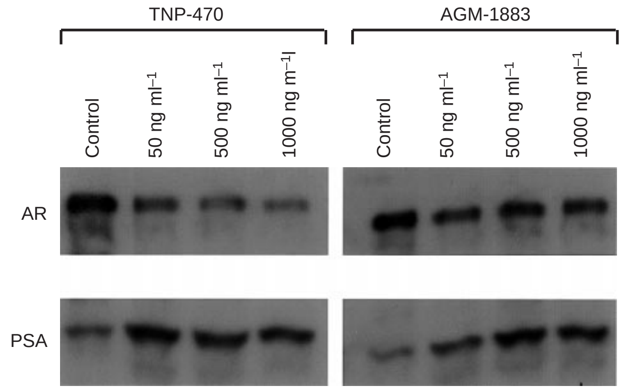

**Figure 3** Immunoblots detecting PSA and AR in LNCaP cell extracts treated with TNP-470 or AGM-1883 at 50, 500 and 1000 ng ml–1. There was an increase in PSA production with both agents following exposure for 120 h. In contrast, AR shows a slight down-regulation which may be indicative of destabilization of the receptor

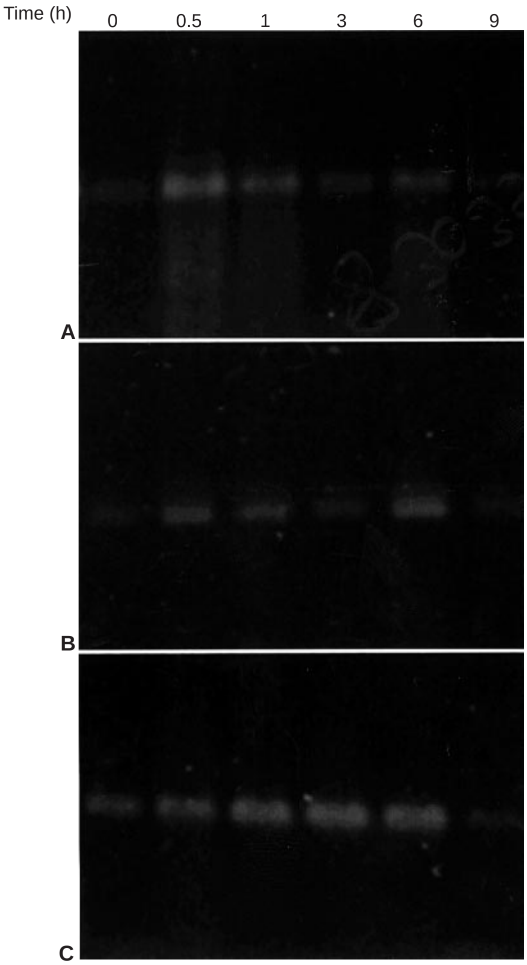

**Figure 4** PCR amplification of cDNA isolated from LNCaP cells treated with 50 ng ml–1 TNP-470. Each cDNA was amplified in duplicate for (**A**) PSA, 540 bp; (**B**) AR, 500 bp; or (**C**) GAPDH, 454 bp. Equivalent volumes of each PCR product was loaded

### MATERIALS AND METHODS

### Cell culture

The human androgen-dependent prostate carcinoma cell line, LNCaP, was obtained from the American Type Culture Collection (Manassas, VA, USA) and grown as directed. Cells, passage 25, were plated at 30 000 cells per 2-cm well. TNP-470 and AGM-1883 were gifts from the Takeda Pharmaceutical Ltd (Oseka, Japan) and prepared as stock solutions in dimethyl sulfoxide (DMSO). Serial dilutions were prepared in culture medium to produce final concentrations of 50, 500 and 1000 ng ml–1. The final DMSO concentration was 0.5%. Cells were treated daily.

### PSA quantification

Every 24 h, the supernatants were collected. Adherent cells were trypsinized and counted on a Coulter Z1 counter (Coulter Electronics, Hialeah, FL, USA). PSA secreted into the supernatant was measured using the Tandem-E PSA assay (Hybritech, San Diego, CA, USA) according to the company’s instructions. The amount of PSA secreted per cell was calculated for every 24 h period.

### Immunoblots for PSA and AR

Following treatment for 120 h, adherent cells were collected by trypsinization. The cells were washed with phosphate-buffered saline and pelleted. Cytosolic protein was extracted by resuspending cells in 10 mM HEPES, pH 7.4, 1 mM EDTA and protease inhibitors (completeØ, Boehringer-Mannheim, Indianapolis, IN, USA) followed by three cycles of freezing and thawing. Cellular debris was pelleted and the supernatant saved. The amount of protein was determined using the BioRad protein assay (BioRad, Hercules, CA, USA). Equal amounts of protein were separated on polyacrylamide-SDS gels, then transferred to nitrocellulose. Blots were developed according to Western-Light chemiluminescent detection system (Tropix, Bedford, MA, USA) using a mouse anti-PSA (Cappel, Aurora, OH, USA) or a mouse anti-human androgen receptor (AR) antibody (Pharmingen, San Diego, CA, USA). The secondary antibody was alkaline phosphatase-conjugated goat anti-mouse IgG (Promega, Madison, WI, USA).

### Reverse transcriptase-polymerase chain reaction assay

LNCaP cells, passage 26, were plated at a density of 45 000 cells per 2-cm well. After 48 h, the cells were treated with 50 ng ml–1 TNP-470. At each time-point, the supernatant was aspirated and the adherent cells lysed in TriZol (Life Technologies, Gaithersburg, MD, USA). Total RNA was extracted according to the manufacturer’s protocol. Contaminating genomic DNA was digested with DNAse I (Ambion, Austin, TX, USA) and first strand cDNA was prepared using an oligo(dT) primer and Superscript™ II reverse transcriptase (Life Technologies, Gaithersburg, MD, USA). Each cDNA:RNA hybrid was digested with RNase H. The final cDNA product was diluted threefold in 10 mM Tris-HCl, pH 7.4, 1 mM EDTA.

PSA and AR primers were synthesized by BioServe Biotechnologies (Laurel, MD, USA) and are as follows: PSA 614, 5′-ATGAGCCTCCTGAAGAATCGATTCCTC-3′; PSA 1154, 5′-AGTCTTGGCCTGGTCATTTCCAAGGT-3′; AR 2381, 5′-TGGGGCTCATGGTGTTTG-3′; and AR 2881, 5′-CAGAAAG-GATCTTGGGCAC-3′. Primers are numbered to correspond to the base in the published sequence to where the primers anneal. HMR GAPDH polymerase chain reaction (PCR) primers were purchased

 © Cancer Research Campaign 1999

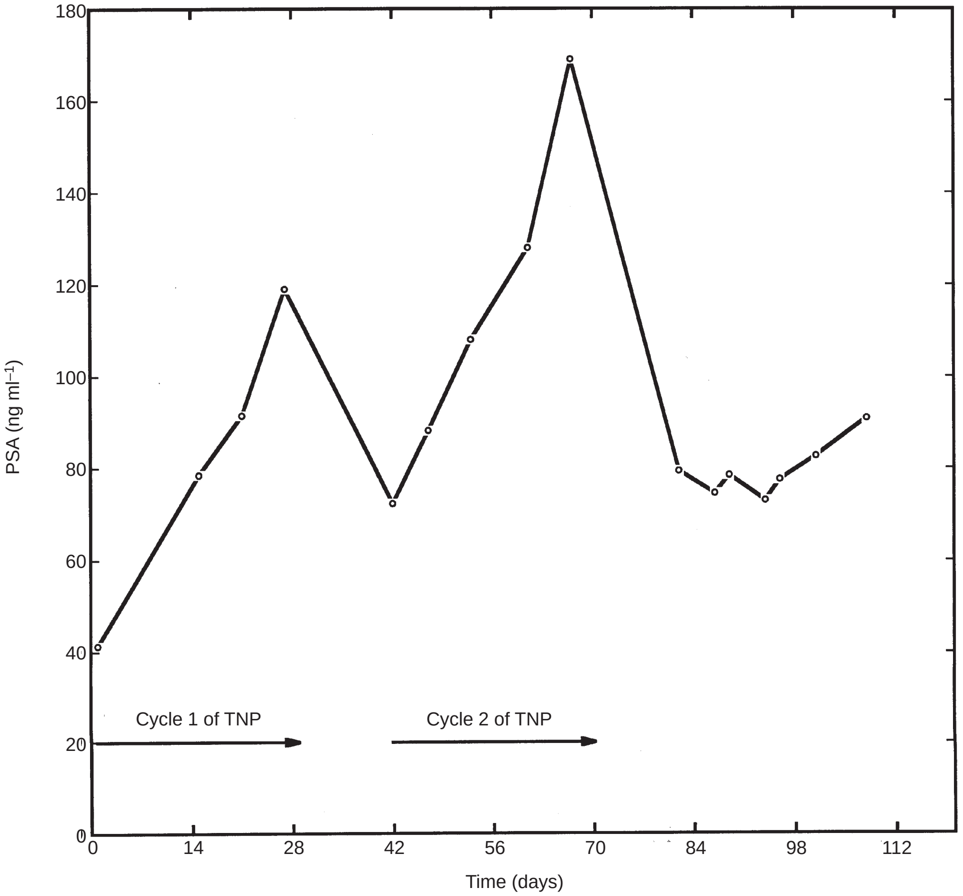

**Figure 5** A patient with Stage D2 AIPC who received two cycles of TNP-470 (over 4 weeks separated by a 2-week break) had a rise in his PSA while receiving therapy. His PSA declined with the discontinuation of treatment and rose slowly after being removed from study from Clontech (Palo Alto, CA, USA). For each pair of primers, the sequence to be amplified was subcloned into pCR-Script™ AMP SK(+) (Stratagene, San Diego, CA, USA) to serve as a positive control. PCR was performed on 2 ng of positive control or 1 µl diluted cDNA in the presence of 50 mM Tris-HCl, pH 8.3, 250 µg $ml^{-1}$ bovine serum albumin, 2 mM magnesium chloride for AR or 3 mM magnesium chloride for PSA and GAPDH, 0.5% Ficoll 400, 1 mM tartrazine, 500 µM of each dNTP, 1 µM of each primer, and 0.5 U Ampli*Taq* (Perkin-Elmer, Foster City, CA, USA) in a volume of 10 µl. Thermal cycling parameters for each primer set were optimized fall within a linear amplification range. The thermal cycling programme consisted of denaturation at 94°C for 15 s followed by several cycles of denaturation at 94°C for 0 s, annealing at the optimized temperature for 0 s, and extension at 72°C for 30 s. The cycle number and annealing temperature for the three primer sets were: 27 cycles and 60°C for PSA; 29 cycles and 55°C for AR; and 22 cycles and 50°C for GAPDH. PCR products (7 µl) were separated on agarose gels. The gels were stained with SYBR Green I (Molecular Probes, Eugene, OR, USA) and scanned on a Fluorimager SI (Molecular Dynamics, Sunnyvale, CA, USA). Bands were quantitated using NIH Image 1.6. Differences in RNA isolation and cDNA synthesis efficiencies between time-points and duplicates were accounted for by normalization of each band to the level of GAPDH detected at the same time-point. Induction was determined relative to the initial time-point.

### RESULTS

After 120 h, TNP-470 treatment resulted in a 40.6% decrease in cell number at the highest concentration (Figure 1A). The mean number of cells per well was $3.82 \times 10^4$ at 50 ng ml, $4.47 \times 10^4$ at 500 ng $ml^{-1}$, and $4.65 \times 10^4$ at 1000 ng $ml^{-1}$ TNP-470. The mean cell number in control wells was $7.83 \times 10^4$. AGM-1883 had a minimal effect on cell viability resulting in a 16.6% decrease in cell number at the highest concentration (Figure 1B). After 120 h, there were $3.84 \times 10^4$, $4.74 \times 10^4$, and $3.47 \times 10^4$ cells/well at 50, 500, and 1000 ng $ml^{-1}$, respectively, compared to $4.16 \times 10^4$ cells/well for controls.

TNP-470 treatment resulted in a 1.1- to 1.5-fold induction in PSA secretion per cell that was concentration-dependent (Figure 2A). Lower concentrations of TNP-470 up-regulated PSA secretion per cell better than higher concentrations. The concentration of PSA in the supernatant at 120 h was 2616.5 fg/cell at 50 ng $ml^{-1}$, 2055.9 fg/cell at 500 ng $ml^{-1}$, 1921.5 fg/cell at 1000 ng $ml^{-1}$ versus 1716.6 fg/cell for the controls. The same trends were observed for AGM-1883 with the amount of PSA secreted per cell up-regulated approximately 30–70% compared to controls (Figure 2B). However, no concentration-dependency was observed in response to AGM-1883. Immunoblots of cell extracts treated with TNP-470 or AGM-1883 for 120 h showed equivalent increases in intracellular PSA (Figure 3). The AR, on the other hand, shows a slight down-regulation in intracellular protein levels relative to

© Cancer Research Campaign 1999

control (approximately 10% decrease). This decrease may be due to instability of the AR protein in the presence of these agents.

The effect of TNP-470 on PSA and AR transcription was quantitated using RT-PCR. Both PSA and AR showed a transcriptional up-regulation that peaked within 30 min of TNP-470 exposure then declined to baseline levels by 9 h (Figure 4). PSA transcription was increased 1.4-fold at 30 min, while AR transcription was induced 1.2-fold. The increased transcriptional activity from the PSA gene accounts for the increased amount of PSA secreted per cell. Increased AR transcription was reflected by similar increases at the protein level.

### DISCUSSION

TNP-470, a semi-synthetic derivative of fumagillin, was discovered by Ingber et al (1990) to inhibit capillary endothelial cell growth. Figg et al (1994, 1997*b*) characterized the pharmacokinetic profile of TNP-470 obtained as part of a dose escalation phase I trial. There was a linear relationship between TNP-470 dose and $AUC_{(inf)}$ and between TNP-470 dose and peak plasma concentrations ($C_{max}$). The $C_{max}$ ranged from 6.6 ng $ml^{-1}$ at the lowest dose level to 597.1 ng $ml^{-1}$ at the highest dose level. TNP-470 was rapidly cleared from the circulation with a short terminal half-life (0.88 ± 2.5 h) consistent with preclinical data. The Cmax of AGM-1883, an active metabolite, ranged between 0.4 to 158.1 ng $ml^{-1}$. Concentrations of TNP-470 that yielded in vitro activity were clinically achievable. In our in vitro experiments, TNP-470 causes an initial decrease in LNCaP cell number at 48 h. Subsequently, cell number was constant throughout the remainder of the treatment period. Cytotoxicity was not noted. AGM-1883 also showed an initial decrease in cell number compared to the control. However, the cell number continued to increase throughout the treatment period, but with a slower doubling time than untreated cells. Thus, it appears that TNP-470 has a slightly stronger cytostatic activity than its metabolite.

Logothetis et al (1997) reported the maximum tolerated dose of TNP-470 to be 70.8 mg $m^{-2}$ and the dose-limiting toxicity as a reversible neuropsychiatric symptom complex (asthenia, ataxia, agitation). They were unable to document any anti-tumour activity in their phase I study, but did note a transient stimulation of serum PSA in a subset of patients. In fact, between 33% and 100% of patients at each dose level had > 50% decline in PSA on two consecutive measurements after cessation of therapy. A case report of a single patient treated with TNP-470 illustrates the dilemma of alterations in PSA independent of anti-tumoral activity may result (Logothetis et al, 1997). In this instance, the patient had a rise in his serum PSA concentration while receiving therapy, followed by a decline in PSA with the discontinuation of TNP-470 (Figure 5). The apparent dissociation between PSA levels and tumour progression in this case and the experience reported by the MD Anderson Cancer Center (unpublished) suggests that this phenomena of PSA expression is reflected clinically.

Our data show that TNP-470 results in a moderate to substantial increase in PSA secreted per cell that is concentration-dependent. Equivalent increases in intracellular PSA were also detected. The active metabolite, AGM-1883, also increased PSA production. However, the increase in PSA secretion in response to AGM-1883 treatment was not concentration-dependent. The increased transcription activity from the PSA gene accounts for the increased amount of PSA produced and secreted by the cell. Increased AR protein could also be accounted for by corresponding increases in AR transcription. Thus, it appears that the mechanism of PSA up-regulation by TNP-470 is at the transcriptional level. For individuals with prostate cancer receiving TNP-470, these data suggest that PSA is not a reliable surrogate marker.

### ACKNOWLEDGEMENTS

We would like to express our appreciation for the continued support and advice of Dr Judah Folkman in the attempt to develop agents that target angiogenesis inhibition for the treatment of cancer.

This work was supported in part by an unrestricted gift from the F. J. McGuigan Trust, and the Visiting Scholars Program of the EORTC.

### REFERENCES

Abe J, Zhou W, Takuwa N, Taguchi J, Kurokawa K, Kumada M and Takuwa Y (1994) A fumagillin derivative angiogenesis inhibitor, AGM-1470, inhibits activation of cyclin-dependent kinases and phosphorylation of retinoblastoma gene product but not protein tyrosyl phosphorylation or protooncogene expression in vascular endothelial cells. *Cancer Res* **54**: 3407–3412

Bauer KS, Dixon SC and Figg WD (1998) Inhibition of angiogenesis by thalidomide requires metabolic activation with is species-dependent. *Biochem Pharmacol* **55**: 1827–1834

Figg WD, Yeh HJC, Thibault A, Pluda JM, Itoh F, Yarchoan R and Cooper MR (1994) Assay of the antiangiogenic compound, TNP-470, and one of its metabolites, AGM-1883, by reversed-phase high performance liquid chromatography in plasma. *J Chrom* **652**: 187–194

Figg WD, Ammerman K, Patronas N, Steinberg SM, Walls RG, Dawson N, Reed E and Sartor O (1996) Lack of correlation between prostate-specific antigen and the presence of measurable soft tissue metastases in hormone-refractory prostate cancer. *Cancer Invest* **14**: 513–517

Figg WD, Bergan R, Brawley O, Tompkins A, Linehan M, Duray P, Bauer KS, Pluda J and Reed E (1997*a*) Randomized phase II study of thalidomide in androgen independent prostate cancer. *Proc Am Soc Clin Oncol* **16**: 1235

Figg WD, Pluda JM, Lush RM, Saville MW, Wyvill K, Reed E and Yarchoan R (1997*b*) The pharmacokinetics of TNP-470, a new angiogenesis inhibitor. *Pharmacotherapy* **1**: 91–97

Folkman J and Ingber D (1992) Inhibition of angiogenesis. *Semin Cancer Biol* **3**: 89–96

Ingber D, Fujita T, Kishimoto S, Sudo K, Kanamary T, Brem H and Folkman J (1990) Synthetic analogues of fumagillin that inhibit angiogenesis and suppress tumour growth. *Nature* **348**: 555–557

Inoue K, Korenagea H, Tanaka N, Sakamoto N and Kadoya S (1988) The sulfated polysaccharide-peptidoglycan complex potently inhibits embryonic angiogenesis and tumor growth in the presence of cortisone acetate. *Carbohydrate Res* **181**: 135–142

Kato T, Sato K, Kakinuma H and Matsuda Y (1994) Enhanced suppression of tumor growth by combination of angiogenesis inhibitor O-(chloroacetyl-carbamoyl) fumagillol (TNP-470) and cytotoxic agents in mice. *Cancer Res* **54**: 5143–5147

Kelly WK, Scher HI, Mazumdar M, Vlamis V, Schwartz M and Fossa SD (1993) Prostate-specific antigen as a measure of disease outcome in metastatic hormone-refractory prostate cancer. *J Clin Oncol* **11**: 607–615

Kusaka M, Sudo K, Fujita T, Marui S, Itoh F, Ingber D and Folkman J (1991) Potent anti-angiogenic action of AGM-1470: comparison to the fumagillin parent. *Biochem Biophys Res Commun* **174**: 1070–1076

Logothetis CJ, Wu K, Jaeckle KA, Amato R, Finn L, Weiss R, Daliani D, Figg WD, Ghaddar H and Gutterman J (1997) Phase I trial of the angiogenesis inhibitor TNP-470 for progressive androgen-independent prostate cancer. *Clin Cancer Res* (submitted)

Lush RM, Figg WD, Pluda JM, Bitton R, Headlee D, Kohler D, Reed E, Sartor O and Cooper MR (1996) A phase I study of pentosan polysulfate sodium in patients with advanced malignancies. *Ann Oncol* **7**: 939–944

Maione TE, Gray GS, Petro J, Hunt AJ, Donner AL, Bauer SI, Carson HF and Sharpe RJ (1990) Inhibition of angiogenesis by recombinant human platelet factor-4 and related peptides. *Science* **247**: 77–79

Masiero L, Figg WD and Kohn EC (1997) New anti-angiogenesis agents: review of experience with CAI, thalidomide, TNP-470, and IL-12. *Angiogenesis J* **1**: 23–35

Matsubara T, Saura R, Hirohata K and Ziff M (1989) Inhibition of human endothelial cell proliferation in vitro and neovascularization in vivo by D-penicillamine. *J Clin Invest* **83**: 158–167

 © Cancer Research Campaign 1999

O'Reilly MS, Brem H and Folkman J (1995) Treatment of murine hemangioendotheliomas with the angiogenesis inhibitor AGM-1470. *J Pediatr Surg* **30**: 325–329

Sartor O (1995) Prostate-specific antigen changes before and after administration of an angiogenesis inhibitor (TNP-470). *Oncol Rep* **2**: 1101–1102

Sridhara R, Eisenberger MA, Sinibaldi V, Reyno LM and Egorin MJ (1995) Evaluation of prostate-specific antigen as a surrogate marker for response of hormone-refractory prostate cancer to suramin therapy. *J Clin Oncol* **13**: 2944–2953

Taylor S and Folkman J (1982) Protamine is an inhibitor of angiogenesis. *Nature* **297**: 307–312

Thalmann GN, Sikes RA, Chang SM, Johnston DA, von Eschenbach AC and Chung LW (1996) Suramin-induced decrease in prostate-specific antigen expression with no effect on tumor growth in the LNCaP model of human prostate cancer. *J Natl Cancer Inst* **88**: 794–801

Thibault A, Sartor O, Cooper MR, Figg WD and Myers CE (1993) A 75% decline in prostate specific antigen (PSA) predicts survival in hormone refractory prostate cancer. *Proc Am Assoc Cancer Res* **34**: A1143

Thorpe PE, Derbyshire EJ, Andrade SP, Press N, Knowles PP, King S, Watson GJ, Yang YC and Rao-Bette M (1993) Heparin-steroid conjugates: new angiogenesis inhibitors with antitumor activity in mice. *Cancer Res* **53**: 3000–3007

Voest EE, Kenyon BM, O'Reilly MS, Truitt G, D'Amato RJ and Folkman J (1995) Inhibition of angiogenesis in vivo by interleukin 12. *J Natl Cancer Inst* **87**: 581–586

Walls R, Thibault A, Lei L, Wood C, Kozlowsky JM, Figg WD, Sampson ML, Elin RJ and Samid D (1996) The differentiating agent phenylacetate increases prostate-specific antigen production by prostate cancer cells. *Prostate* **29**: 177–182

Wasilenko WJ, Palad AJ, Somers KD, Blackmore PF, Kohn EC, Rhim JS, Wright GL Jr and Schellhammer PF (1996) Effects of the calcium influx inhibitor carboxyamido-triazole on the proliferation and invasiveness of human prostate tumor cell lines. *Int J Cancer* **68**: 259–264

*© Cancer Research Campaign 1999*

